# Direct Experimental Comparison of Liposome Formulation Processes for Poorly Permeable Solutes: Effect of Driving Force and Bilayer Disruption Mechanism

**DOI:** 10.1021/acsomega.5c09893

**Published:** 2026-04-16

**Authors:** Martin Roudný, Martin Balouch, Jaroslav Hanuš, František Štěpánek

**Affiliations:** 1 Department of Chemical Engineering, 534467 University of Chemistry and Technology Prague, Technická 5, Prague 6 166 28, Czech Republic; 2 Zentiva, k.s., U.K.abelovny 130, Prague 10 102 37, Czech Republic

## Abstract

Liposomes can act as drug carriers because of their ability to encapsulate both hydrophilic and lipophilic substances, protect them from external factors, and facilitate controlled drug delivery or release. However, the physical properties and encapsulation efficiencies are strongly influenced by the choice of the liposome formation and the solute loading process. During liposome assembly, poorly permeable solutes can be rejected by the phospholipid membrane, resulting in a poor encapsulation efficiency. In this study, five different liposome formulation process routes have been directly experimentally compared: the heating method with sonication, the lipid film hydration method with either extrusion or sonication, and the freeze–thawing method applied to either preloaded or blank liposomes. 5(6)-Carboxyfluorescein and D-(+)-glucose were used as model solutes with different permeability and hydrophilicity profiles to assess the encapsulation efficiency and concentration retention factor of each formulation process route. The film hydration method followed by extrusion was found to be the most effective for carboxyfluorescein as a more permeable solute. In contrast, the film hydration method followed by sonication was found to be a more effective process for glucose, which is less permeable. The quantity of encapsulated solute per mass of lipids was found to increase with an increasing solute concentration in the loading solution, indicating a clear dependence on the concentration driving force.

## Graphical abstract

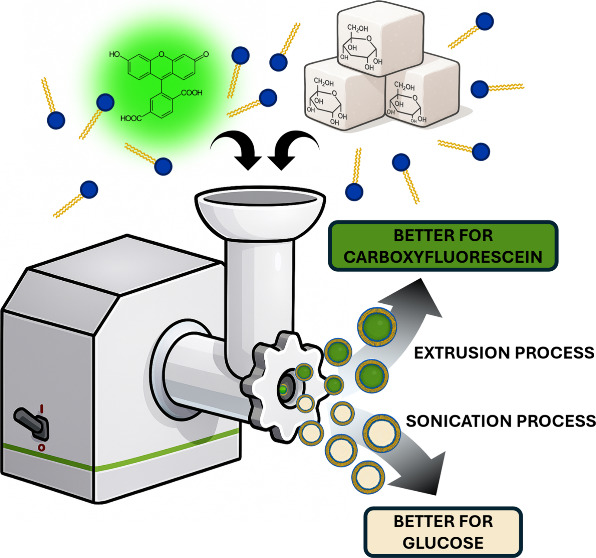

## 1 Introduction

Liposomes, first described by Bangham and Horne in the 1960s,^1^ have gradually transitioned from basic experimental tools to industrially manufactured carriers for nutraceutics and pharmaceutics.^2^ Owing to membrane composition based on amphiphilic phospholipids, liposomes have the ability to encapsulate either hydrophilic molecules into their aqueous core or lipophilic substances within the lipidic membrane.^3^ Examples of clinically relevant liposomal formulations include the anticancer drug Doxil,^4^ various antifungal agents, formulations targeting the respiratory system, and vaccine delivery systems.^5^ Liposomes can provide protection, prolonged bloodstream circulation, and controlled release of the encapsulated bioactive compounds.^6–8^ This versatility is crucial in addressing formulation challenges, particularly for chemically unstable or toxic actives. However, liposomal formulations still represent a relatively small fraction of newly approved drugs, reflecting the challenges associated with the efficiency and scalability of the manufacturing process.^9^

The incorporation of bioactive payloads into liposomes depends not only on the lipid bilayer composition but also on the chosen liposome formation process route, which can influence the final size distribution, lamellarity, and encapsulation efficiency.^5^ Various methodologies have been developed for liposome production, for example, the high-pressure methods,^10,11^ the ethanol injection method, the reverse-phase evaporation method, the heating method, or the lipid film hydration method. The lipid film hydration method enables the encapsulation of both hydrophilic and hydrophobic substances and generally necessitates subsequent liposome size adjustment by sonication or extrusion.^12,13^ The main drawbacks of this method include limited scalability, the necessity of postprocessing steps, and variable encapsulation efficiency, caused by the need for the solute to permeate through the nascent lipid bilayer during the hydration and budding of the lipid film. The ethanol injection method presents a more scalable approach that is less dependent on the solute permeability but results in diluted liposomes with a residual solvent in the lipid membrane.^14,15^ A higher encapsulation efficiency can generally be achieved by the reverse-phase evaporation method, whose downside is the use of organic solvents along with time-consuming and energy-intensive purification process steps.^16,17^ An alternative to solvent exchange approaches is the heating method (also called the Mozafari method), where liposome assembly takes place purely in the aqueous phase, and solute loading is achieved by heating the system well above the phase transition temperature of the lipid bilayer to increase the permeation rate. As all process steps occur in bulk, a relatively simple scale-up is possible.^18,19^

Apart from the formation of “raw” lipidic vesicles, the adjustment of the liposome size and polydispersity may be required as an additional process step. Size distribution is crucial especially for injectables, whereas the demands on size uniformity in liposomal formulations intended for oral delivery are less strict.^20^ Size adjustment can be achieved for example by sonication or membrane extrusion.^11^ Repeated lipid bilayer stretching, rupture, and fusion that occurs during these processes can also affect the final drug encapsulation efficiency. Therefore, an understanding of the advantages and disadvantages of various process routes for liposome production is crucial to enable rational process choices for each specific drug and therapeutic application. There is an absence of clear guidelines matching specific formulation process routes and membrane composition to individual substances and the transition from small-scale fabrication methods to industrial production.^21–23^ Each drug poses individual drug–lipid interaction and physicochemical properties, including lipophilicity, charge, or permeability, all of which influence the encapsulation efficiency of the drug in a chosen manufacturing process.^24–27^

Although in principle, many studies on drug encapsulation into liposomes can be found in the literature, the conditions (e.g., lipid bilayer composition, nature of the encapsulated solute, temperature, pH, etc.) are rarely identical. This makes it virtually impossible to carry out a direct comparison of the individual process options purely based on published data. Therefore, the present study focuses on a direct experimental comparison of five different liposome formulation process routes using identical lipid and buffer compositions and compares the encapsulation efficiency of two model poorly permeable solutes, as such solutes pose a particular challenge for liposome encapsulation. Specifically, attention is paid to the role of membrane rejection, whereby the solvent (water) can cross the lipid bilayer easily, while the poorly permeable solute cannot. The liposome formulation process routes, summarized in Figure 1, were as follows: the heating method followed by sonication, the lipid film hydration method followed by either extrusion or sonication, and the freeze–thawing method applied to either preloaded liposomes or to blank liposomes with postloading.

**Figure 1:**
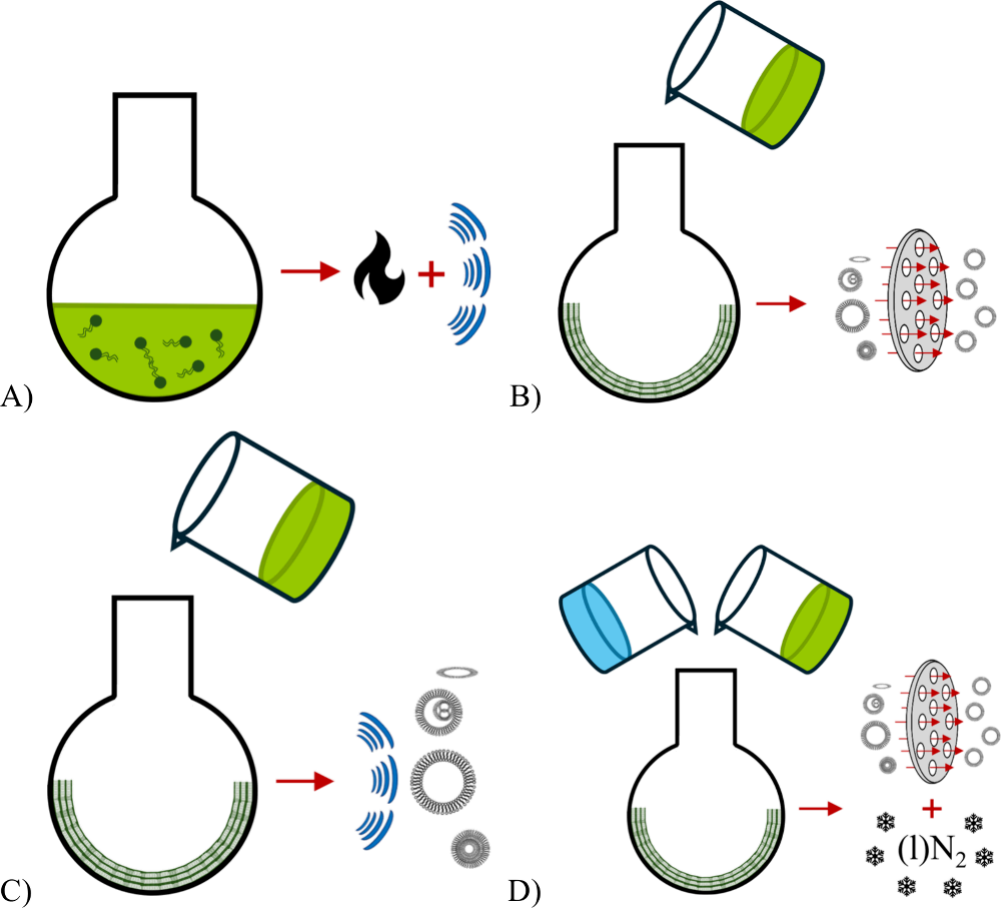
Schematic illustration of the liposome formulation process routes used in this work. (A) Heating with sonication; (B) film hydration followed by extrusion; (C) film hydration followed by sonication; (D) freeze–thawing applied to preloaded or blank liposomes.

Two poorly permeable model substances 5(6)-carboxyfluorescein and D-(+)-glucose were chosen for their structural similarity to small-molecule drugs yet different physicochemical properties, particularly their water solubility and water–lipid partition coefficients. Recently, a classification mechanism of small-molecule drugs based on their liposome membrane permeability and water–lipid partitioning has been proposed.^28,29^ The rationale behind choosing carboxyfluorescein and glucose as the model solutes for the present work stems from this classification system. Based on the partitioning coefficient (log*K*, Table 1), carboxyfluorescein is likely to be located both in the lipidic membrane and the aqueous cavity of liposomes, while glucose will tend to be present only in the aqueous phase. Although both substances can be classed as poorly permeable, the permeability of glucose is still about 1 order of magnitude lower than that of carboxyfluorescein (logPerm, Table 1).

**Table 1:**
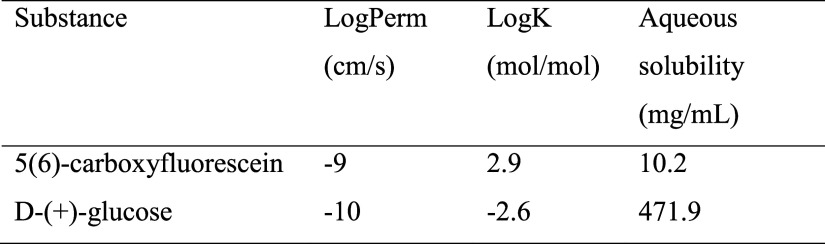
Key Physicochemical Properties^28,30–33^ of Solutes Used for Liposome Encapsulation in This Work^a^

a LogPerm is a measure of permeability through the liposome membrane; log *K* is a measure of the membrane/water equilibrium partition coefficient.

To the best of our knowledge, this study represents the most consistent direct experimental comparison of liposome formulation process routes for poorly permeating solutes. If machine-learning algorithms are to be practically useful for pharmaceutical process development in the future, it is crucial that they be trained on self-consistent data sets such as the one hopefully presented here.

## 2 Materials and Methods

### 2.1 Materials

5­(6)-Carboxyfluorescein [CAS: 72088–94–9] (>95%) (further denoted CF), D-(+)-glucose [CAS: 50–99–7] (>99.5%) (further denoted GLU), cholesterol [CAS: 57–88–5] (>99%), phosphate-buffered saline (PBS) in tablets, and TRITON X-100 [CAS: 9036–19–5] were purchased from Sigma-Aldrich/Merck Life Science s.r.o. (Prague, Czech Republic). DPPC [CAS: 63–89–8] (>98%) and DPPG [CAS: 67232–81–9] (>95%) were purchased from Corden Pharma (Basel, Switzerland). 1-Oleoyl-2-[12-[(7-nitro-2–1,3-benzoxadiazol-4-yl)­amino]­dodecanoyl]-*sn*-Glycero-3-phosphocholine [CAS: 190792–36–0] (NBD PC, > 99%) was purchased from Avanti Polar Lipids (Alabaster, USA). Phosphoric acid [CAS: 7664–38–2] (H_3_PO_4_, > 75%), sodium hydroxide [CAS: 1310–73–2] (NaOH, p.a.), methanol [CAS: 67–56–1] (>99.8%), and sulfuric acid [CAS: 7664–93–9] (H_2_SO_4_, 96%) were purchased from PENTA chemicals s.r.o. (Prague, Czech Republic). Chloroform [CAS: 67–66–3] (p.a.) and ethanol [CAS: 64–17–5] (>96%) were purchased from Lach:Ner s.r.o. (Neratovice, Czech Republic). Deionized water (Aqual 25, 0.07 μS/cm) was used in all experiments.

### 2.2 Liposome Formulation Process Routes

#### 2.2.1 Film Hydration Process Route

Liposomes were prepared following the Bangham’s lipid film hydration method^34^ as follows. A mixture of DPPC, DPPG, and cholesterol (10 mg in 75:10:15 molar ratio) was dissolved in 2 mL of chloroform:methanol equimolar mixture followed by solvent removal using a rotary evaporator (BÜCHI Rotavapor R-100) under reduced pressure (55 °C, constant pressure reduction from atmospheric to about 100mbar at 300 rpm) until the complete removal of a solvent and formation of a lipid film on the wall of the flask (25 mL flask of 4 cm diameter). After complete drying in a desiccator for 24 h, the dried lipid film was hydrated by 2 mL of aqueous medium (0.01 M PBS, CF or GLU solution at 7.5 mg/mL, pH 7.4). In the case of glucose, also solutions with concentrations of 25, 50, and 100 mg/mL (pH 7.4) were tested. The formed raw vesicles were extruded 21 times through a polycarbonate membrane (Avanti Mini Extruder, pore size 200 nm) at 70 °C or sonicated (Ultrasonic cleaning unit Elmasonic P 30 H, operated at pulse mode at a frequency 37 kHz and power 80 W for 60 min) at 45 °C to form liposomes. The number of passes through the extrusion membrane was shown in our earlier work^46^ to result in unilamellar liposomes whose particle size no longer changes with an increasing number of extrusion steps. The duration of the sonication step was set to reach a state where the size distribution of the liposomes no longer changed. After the extrusion or sonication step, liposomes were maintained at a laboratory temperature to avoid spontaneous leakage of the encapsulated solutes. It has been shown earlier^46^ that the phase transition temperature of a lipid bilayer for the composition considered in the present work is 41.5 °C.

#### 2.2.2 Heating Process Route

Liposomes were prepared by the heating method without using organic solvents, in the literature called the Mozafari method.^35^ A mixture of DPPC, DPPG, and cholesterol (molar ratio 75:10:15) in a powder form was dispersed in a PBS solution containing CF or GLU (7.5 mg/mL, pH 7.4) for 60 min at room temperature followed by heating at 75 °C for an additional 60 min and cooling to room temperature. In the case of glucose, also solutions with concentrations of 25, 50, and 100 mg/mL (pH 7.4) were tested. The formed raw vesicles were subsequently sonicated until no further change of the liposome particle size distribution was observed (30 min at 45 °C). Thus, the final size distribution of the liposomes represents an asymptotic state under these process conditions.

#### 2.2.3 Freeze–Thawing Process Route

During the growth of raw liposomes formed by the hydration of a lipid film, poorly permeable solutes can, in principle, be rejected from the budding vesicles as only the solvent (water) can cross the lipid bilayer. To assess the extent to which this phenomenon might play a role in liposome loading by each of the two solutes considered in this work, freeze–thaw cycles were applied to liposomes prepared by the film hydration method that were either blank (hydration by solute-free PBS) or “preloaded” (hydration by 7.5 mg/mL of either carboxyfluorescein or glucose). Freeze–thaw cycles are known to temporarily disrupt the lipid bilayer and thus, in principle, allow even poorly permeable solutes to diffuse into the liposomes. Blank liposomes were prepared by the film hydration method followed by extrusion as described above, whereby the dried lipid film was hydrated by 2 mL of aqueous medium (PBS, pH 7.4) not containing any solute followed by 21 cycles of extrusion of the formed raw vesicles through a polycarbonate membrane (pore size 200 nm). 2 mL portion of blank liposomes prepared in this way was then mixed with 2 mL of CF or GLU in an aqueous medium (PBS, pH 7.4) to achieve a final concentration of CF or GLU of 7.5 mg/mL. This mixture underwent five freeze–thaw cycles, consisting of freezing in liquid nitrogen (5 min) and then thawing in a water bath (70 °C, 5 min). As a reference, the combination of the lipid film hydration method with freeze–thawing cycles was performed also for “prefilled” liposomes, i.e., liposomes formed by the lipid film hydration method followed by extrusion, whereby a CF or GLU solution (7.5 mg/mL) was employed already during the film hydration stage instead of the solute-free buffer. This was followed by extrusion and subsequent treatment of the loaded liposomes by freeze–thaw cycles with the same parameters as described above.

All preparations were performed in biological triplicate. The process parameters for all liposome formation routes are summarized in Table 2.

**Table 2:** Summary of Key Parameters of the Liposome Formation and Loading Process Routes

process route	temperature	extrusion cycles	sonication time	freezing cycles
heating with sonication	75 °C (60 min)	n/a	30 min	n/a
film hydration with extrusion	70 °C	21× through 200 nm PC membrane	n/a	n/a
film hydration with sonication	45 °C	n/a	60 min	n/a
freeze–thawing empty liposomes	70 °C	n/a	n/a	5×
freeze–thawing prefilled liposomes	70 °C	n/a	n/a	5×

### 2.3 Purification of Liposomes

To separate liposomes from nonencapsulated solute, all samples were purified using size-exclusion chromatography (Sephadex G-25 Medium). A 0.5 mL sample of liposomes was injected into the column (pore size 5 kDa, PD Miditrap G-25, Cytiva), which was then repeatedly eluted with 0.5 mL of pure aqueous medium (PBS, pH 7.4). 1 mL of purified liposomes was collected from the column (elution time approximately 60 s) to ensure minimal loss of liposomes in the column.

### 2.4 Liposome Characterization

The size distribution of the liposomes was measured by dynamic light scattering using a Zetasizer Nano-ZS (Malvern Instruments, UK). Before measurement, 50 μL of the sample was diluted with 950 μL of PBS (0.01M, pH 7.4). The measurements were run as 3 consecutive sequences (refractive index of liposomes 1.450) in triplicate.

The morphology of liposomes was evaluated by transmission electron microscopy (TEM, Jeol JEM-1010, accelerating voltage of 80 kV).

The phospholipid content of the purified liposomes was determined by ^1^H NMR (Bruker Avance III 500 MHz) according to a simple method for measuring phospholipid terminal alkyls, reported by Hein et al.^36^ This technique allowed for the differentiation of cholesterol from phospholipids but individual phospholipids (DPPC and DPPG) could not be distinguished. However, this does not matter, as there is no reason to expect phase separation of DPPC from DPPG.

Liposome stability was evaluated by dynamic light scattering. Aliquots of 50 μL were collected immediately after liposome preparation, and the particle size distribution was measured. Additional measurements were performed after 1, 4, and 7 days of storage at 4 °C.

### 2.5 Evaluation of Solute Encapsulation

The self-quenching property of CF was used for measuring its encapsulation in the liposomes. The quantity of encapsulated CF was determined by the difference in the fluorescence intensity of purified liposomes (where fluorescence is negligible due to self-quenching of the encapsulated CF) and the intensity after the addition of Triton X-100, which causes total micellization of the system with subsequent release and dilution of all encapsulated CF that results in a sharp rise in fluorescence emission. Fluorescence intensity was measured by the Cary Eclipse Fluorescence Spectrophotometer (Agilent) at an excitation wavelength of 490 nm and emission wavelength of 520 nm (detector voltage set to medium). CF was calculated from the fluorescence intensity using a previously constructed calibration curve.

The concentration of GLU was quantified colorimetrically using an oxidase/peroxidase/*o*-dianisidine Glucose Assay Kit (Sigma-Aldrich). 100 μL of the sample was mixed with 200 μL of the assay reagent and incubated for 30 min at 37 °C. The reaction was then stopped by the addition of 200 μL of 9 N H_2_SO_4_ and the resulting colored product was analyzed by UV–vis spectroscopy at λ = 540 nm. The calibration curve was linear in the range of 4 to 40 μg/mL with an *R* ^2^ ≥ 0.999. The encapsulated amount was calculated from the difference between the amount of GLU in the surrounding purified liposome solution and the amount of GLU in the solution after complete release from the liposomes due to micellization by Triton X-100.

The effectiveness of solute encapsulation was expressed by the concentration retention factor *X*_*c*_, defined by eq 1, as the ratio of the solute concentration achieved inside the liposomes to the bulk concentration of the original loading solution

$$X_{c}=\frac{c_{\mathrm{solute}\;\mathrm{in}\;\mathrm{liposomes}}}{c_{\mathrm{solute}\;\mathrm{in}\;\mathrm{loading}\;\mathrm{solution}}}$$ 1

To calculate the concentration of the encapsulated solute within the liposomes, it is necessary to know the total volume of the liposomal core, which in turn depends on the size liposome distribution and the absolute liposome concentration. These were determined by combining nanoparticle tracking analysis^37^ (NTA, Malvern NanoSight NS 300) and dynamic light scattering (DLS, Malvern Zetasizer). The samples were diluted 16000-fold prior to the NTA measurements. Five 60 s videos were recorded for each sample, with the camera level set to 16. The following settings were used for analysis: detection threshold 5, automatic blur size, automatic maximum track length, and automatic maximum expected particle size. The particle concentration obtained from NTA and the volume-mean liposome size provide the total liposomal volume. The solute concentration can then be calculated from the total liposomal volume and the encapsulation amount according to the following equations:

$$V_{\mathrm{liposomes}}=\frac{N_{\mathrm{liposomes}}\pi d_{\mathrm{liposome}}^{3}}{6}$$ 2

$$c_{\mathrm{solute}\;\mathrm{in}\;\mathrm{liposomes}}=\frac{m_{\mathrm{solute}\;\mathrm{encapsulation}}}{V_{\mathrm{liposomes}}}$$ 3

where *V*_liposomes_ is the total volume of formed liposomes, *N*_liposomes_ is the total number of formed liposomes determined by NTA, *d*_liposomes_ is the volume-mean particle size of the liposomes, and *m*_solute_encapsulation_ is the encapsulated mass determined spectrophotometrically as described above.

Statistical significance was evaluated using unpaired two-tailed Student’s *t* test. Differences were considered statistically significant at *p* < 0.05.

## 3 Results and Discussion

### 3.1 Liposome Size and Morphology

Liposomes prepared by each process route were characterized by dynamic light scattering (Figure 2) and transmission electron microscopy (Figure 3). The heating method produced liposomes with a volume-mean size of 58 nm and a relatively high polydispersity index (PDI = 0.28), indicating a heterogeneous population of vesicles, attributed to the stochastic nature of sonication and the presence of phospholipid residues. TEM images supported these findings, revealing the formation of predominantly small unilamellar vesicles (SUVs) but also residual phospholipid material that had not been incorporated into the lipidic bilayers (Figure 3B). It was observed that approximately 30% of the initial phospholipids and cholesterol remained unused, reflecting a certain inefficiency of the heating method. This loss is primarily attributed to incomplete cholesterol solubilization in the absence of organic solvents. This limitation can be mitigated by preheating the cholesterol dispersion to even higher temperatures to ensure complete melting and molecular dispersion before mixing with the phospholipid phase.^38^

**Figure 2:**
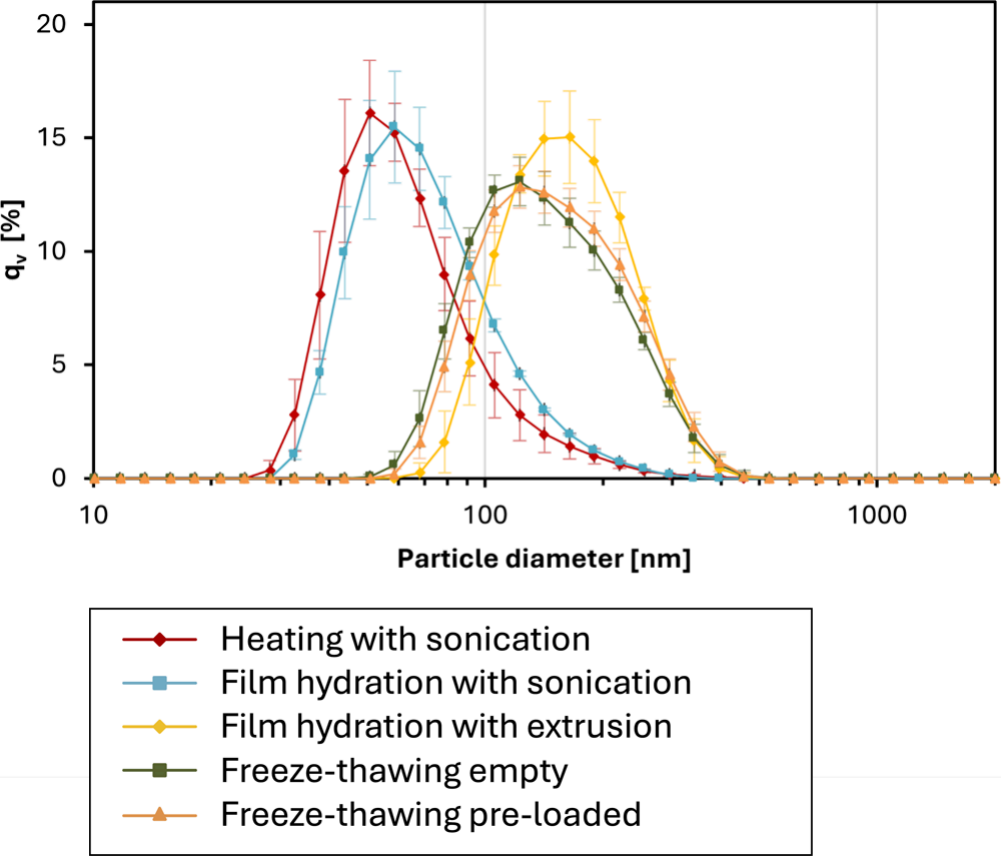
Volume-weighted particle size distributions of liposomes prepared by different process routes (experimental conditions are listed in Table 2), measured by dynamic light scattering (as described in Section 2.4) after liposome loading and purification. The data points are mean values, the error bars represent standard deviations (*N* = 3).

**Figure 3:**
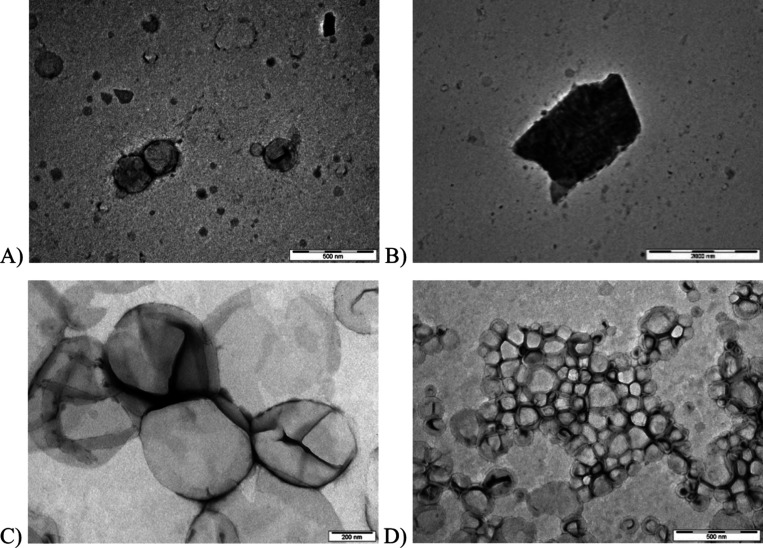
TEM micrographs of liposomes prepared by individual process routes (experimental conditions are given in Table 2). (A) Liposomes prepared by the heating method with sonication (the scale bar represents 500 nm); (B) example of a lipid residue present in samples prepared by the heating method (the scale bar represents 2000 nm); (C) liposomes prepared by the lipid film hydration method followed by extrusion through a 200 nm polycarbonate membrane (the scale bar represents 200 nm); (D) liposomes prepared by the lipid film hydration method followed by sonication (the scale bar represents 500 nm). Additional representative TEM images of these samples can be found in the Supporting Information.

In contrast, the lipid film hydration method followed by extrusion produced liposomes with a more uniform size distribution, defined by the extrusion process. Extrusion through a polycarbonate membrane with a pore size of 200 nm led to the formation of liposomes with a volume-mean particle size of 174 nm and a relatively low PDI of 0.08, indicating a more homogeneous population compared with the heating method. TEM micrographs of the extruded liposomes (Figure 3C) revealed a well-defined morphology, with vesicle sizes closely aligned with the extrusion membrane pore size.

When the raw lipid vesicles formed by lipid film hydration were exposed to sonication rather than extrusion, the resulting liposomes were generally smaller, with a volume-mean size of 63 nm and a PDI of 0.19. This method yielded liposomes of comparable size to those from the heating method. The vesicle population was relatively heterogeneous, presumably due to the random nature of vesicle breakdown and fusion during the sonication process (Figure 3D). The formation of small liposomes by sonication is consistent with the literature,^36^ though the extent of polydispersity is not always fully reported.

The freeze–thaw process route, in which liposomes were first prepared using the lipid film hydration method followed by extrusion and then repeatedly frozen and thawed, produced liposomes with a volume-mean size of 160 nm and PDI of 0.09, which is close to the properties measured before freeze–thawing. It has been previously reported that multilamellar vesicles subjected to freezing–thawing cycles increased their mean diameter with the number of freezing cycles due to the fusion of vesicles.^39^ However, no such behavior was observed in the present work. Since a homogeneous population of small unilamellar vesicles was first prepared by extrusion, the subsequent freezing and thawing had no significant effect on the size distribution and polydispersity compared to simple extrusion as can be seen in Figure 2.

From the weekly stability tests (Figure 4), it has been observed that liposomes prepared by film hydration with extrusion and heating with sonication maintained both their particle size and polydispersity index throughout the 7 day period. In contrast, liposomes processed by film hydration with sonication, which initially displayed the highest polydispersity, showed a slight aggregation indicated by an increase in the mean particle size and the polydispersity index.

**Figure 4:**
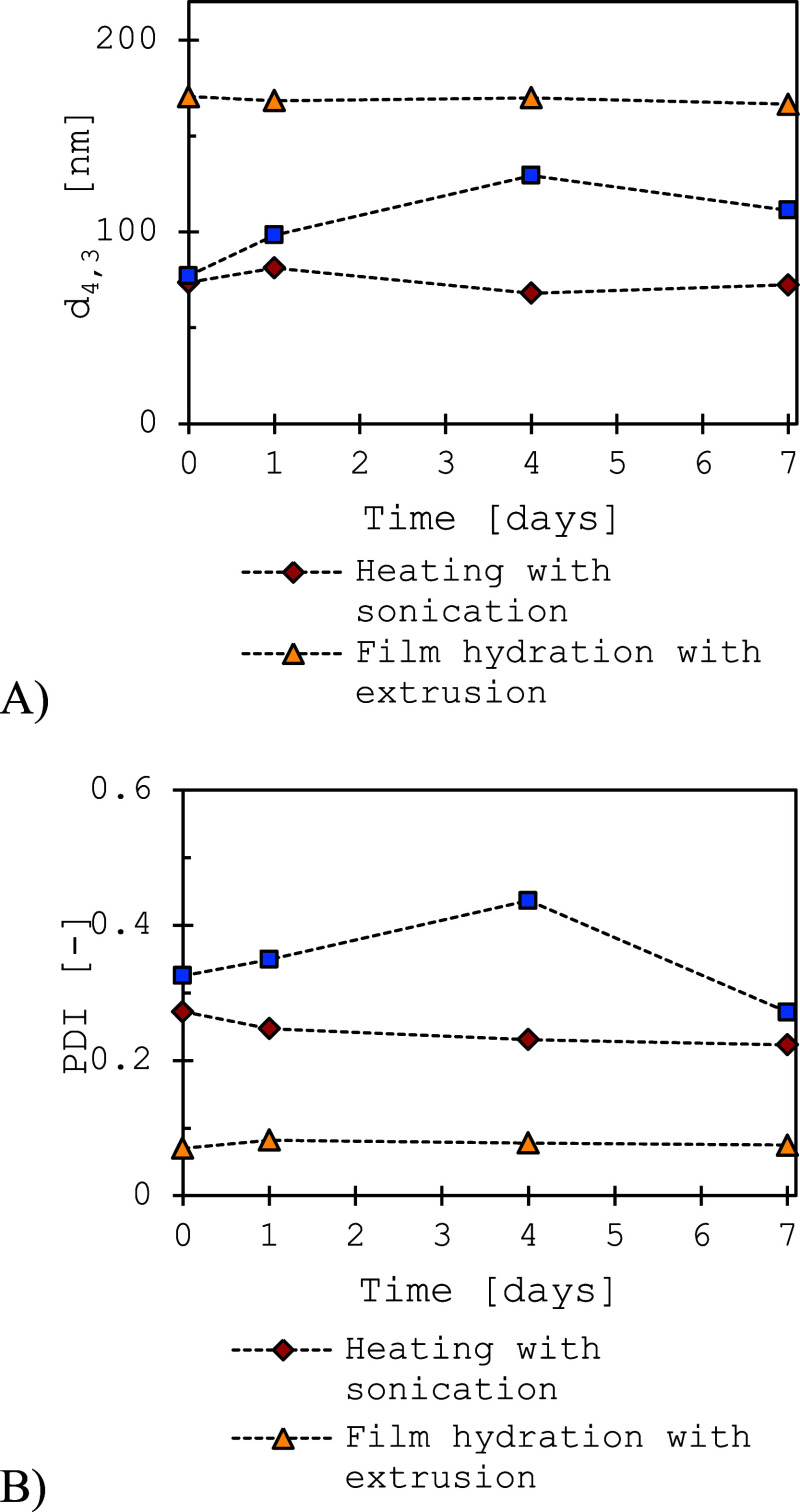
Stability of liposomes over 7 days of storage at 4 °C, prepared by different methods as indicated (experimental conditions are listed in Table 2). (A) Volume-mean particle size; (B) polydispersity index (PDI) of liposomes as a function of time. Detailed numerical values for particle size and PDI over the observed period are provided in Table S1 (Supporting Information).

### 3.2 Composition of the Phospholipid Bilayer

As the composition of the lipid bilayer determines parameters such as phase transition temperature and solute permeability, it is crucial to verify that the individual liposome formulation route conserves the proportion of lipids and cholesterol used at the start of the process. The results are summarized in Table 3. The film hydration method resulted in liposome compositions closely aligned with the nominal phospholipid:cholesterol molar ratio of 85:15, meaning that this method reliably incorporates cholesterol into the bilayer. In contrast, bilayers of liposomes prepared by the heating method were found to contain a significantly reduced cholesterol content (only 3% mol), which means that under the conditions at which the heating method was operated, cholesterol was not fully incorporated into the membrane of liposomes. However, we assume that this compositional deviation should not hinder the encapsulation process since the heating method is based on the spontaneous self-assembly of vesicles. The encapsulation mechanism is therefore driven by the passive entrapment of the bulk aqueous phase as the solute is captured during vesicle closure. After short centrifugation of the liposome suspension prepared by the heating method, the pellet of residues was found to be composed of both phospholipids and cholesterol, but the cholesterol level was elevated (molar ratio 75:25), and it contained approximately 30% of the initial quantity of phospholipids.

**Table 3:** Composition of the Phospholipid Bilayer of Liposomes Prepared by Different Formulation Process Routes^a^

formulation process route	phospholipid molar fraction [%]	cholesterol molar fraction [%]
heating with sonication	97 ± 3	3 ± 3
film hydration with extrusion	82 ± 1	18 ± 1
film hydration with sonication	82 ± 1	18 ± 1

a Experimental conditions are listed in Table 2. The data represent mean values ± standard deviation (*N* = 3).

### 3.3 Comparison of Encapsulation Efficiency

In the following discussion, the extent of solute encapsulation into liposomes is expressed by two values. First, the encapsulated quantity will be expressed as the solute/lipid ratio (S/L ratio), defined as the mass ratio of the total encapsulated solute to the mass of lipids used for the preparation of liposomes and given in μg of solute (carboxyfluorescein or glucose) per milligram of lipids. Second, the concentration retention factor *X*_c_, defined by eq 1, will be used to describe the ratio of solute concentration inside the liposomes to that of the bulk solution used during solute loading. The concentration retention factor *X*_c_ can be regarded as an indirect measure of solute rejection by the liposomal membrane. When a water-soluble substance is not able to cross the lipid bilayer during attempted liposome loading, then *X*_c_ = 0; on the other hand, when the solute concentrations inside and outside of liposomes after loading are equal, then *X*_c_ = 1. Theoretically, *X*_c_ should be independent of the liposome size, whereas the S/L ratio should, in principle, depend on the surface to volume ratio of the liposomes and therefore on their size.

The results for the encapsulation of both solutes by each process route are summarized in Table 4. For carboxyfluorescein, the film hydration method with extrusion achieved a significantly higher loading (S/L ratio = 37 ± 4 μg_CF_/mg_LIP_) compared to the film hydration method with sonication (*p* < 0.05). While extrusion produced liposomes around 200 nm in size, the methods using sonication yielded smaller liposomes (around 60 nm) and a lower S/L ratio for carboxyfluorescein, namely, S/L ratio = 22 ± 6 μg_CF_/mg_LIP_ by the film hydration method with sonication and S/L ratio = 36 ± 9 μg_CF_/mg_LIP_ by the heating method with sonication. A similar trend was previously reported for resveratrol^40^ (logP = 2.97). The freeze–thaw method applied to blank liposomes resulted in a low quantity of caboxyfluorescein encapsulation (S/L ratio = 15 ± 2 μg_CF_/mg_LIP_).

**Table 4:** Comparison of Liposome Formulation Process Routes in Terms of the Achieved Solute/Lipid Ratio (S/L) and the Concentration Retention Factor (*X*_c_) for Both Solutes at Identical Initial Loading Concentration of 7.5 mg/mL^a^

	5(6)-carboxyfluorescein		D-(+)-glucose	
process route	S/L ratio [μg_CF_/mg_LIP_]	*X*_c_ [-]	S/L ratio [μg_GLU_/mg_LIP_]	*X*_c_ [-]
heating with sonication	36 ± 9^b^	1.0	25 ± 8^b^	1.0
film hydration with extrusion	37 ± 4	0.6	14 ± 4	0.25
film hydration with sonication	22 ± 6	0.6	34 ± 5	0.9
freeze–thawing empty liposomes	15 ± 2	0.2	7 ± 4	0.15
freeze–thawing prefilled liposomes	59 ± 8	0.75	17 ± 4	0.3

a Detailed experimental conditions are listed in Table 2. The data represent mean values ± the standard deviation (*N* = 3).

b Approx. 30% of phospholipids remained unused in the preparation of liposomes by the heating method. This leads to a reduction in the total volume of the formed liposomes and consequently lowers the S/L ratio, which is defined using the initial quantity of lipids.

However, this experiment has proved that repeated stressing of the lipid bilayer by the freeze–thaw cycles facilitated at least partial solute diffusion into previously “empty” liposomes. Consequently, the highest quantity of encapsulated carboxyfluorescein was achieved when the freeze–thaw cycles were applied to liposome that were already partially prefilled by carboxyfluorescein during the film hydration and extrusion steps. In this approach, the encapsulated quantity achieved initially was further enhanced by the freeze–thaw cycles and resulted in a final S/L ratio = 59 ± 8 μg_CF_/mg_LIP_, which was a statistically significant increase compared to extrusion alone (*p* < 0.05). Considering the standard deviations, it can be stated that contributions by the two loading methods to the total encapsulated quantity were additive in this case.

A comparison of the concentration retention factors provides a somewhat different view of the encapsulation methods, as it reveals the role of solute permeation across the membrane during loading. The heating method resulted in the highest *X*_c_ = 1.0, since liposomes were formed from lipids in a disordered state directly in the carboxyfluorescein solution and there was no need for the solute to permeate across any existing lipid bilayer. Thus, possible solute rejection by the membrane did not play a role. For the film hydration method, where solute rejection by the budding lipid film may play a role, the final concentration retention factors were lower (*X*_c_ = 0.6). Interestingly, they were the same regardless of whether extrusion or sonication was used for adjusting the liposome size.

Although both methods in principle result in temporary disruption of the lipid bilayer, the extent and duration of this disruption depends on the exact process parameter settings (e.g., sonication intensity and duration, number of extrusion passes, etc.). Thus, it might be just coincidence that the same concentration retention factor was eventually obtained. As expected, subjecting the liposomes to freeze–thaw cycles resulted in an additional solute diffusion into the liposome and the concentration retention factor increased to *X*_c_ = 0.75. Applying the freeze–thaw cycles to blank liposomes resulted in *X*_c_ = 0.2, which again proves that certain membrane permeabilization had occurred during these steps.

Despite maintaining the same process conditions of liposomes as for caboxyfluorescein, the order of the process routes based on glucose encapsulation did not remain the same. The highest solute/lipid ratio as well as the highest concentration retention factors for glucose were obtained by methods utilizing sonication. Even though small liposomes were formed, the film method with sonication encapsulated 34 ± 5 μg_GLU_/mg_LIP_ (*X*_c_ = 0.90), which was significantly higher than the film hydration method with extrusion (*p* < 0.01). The heating method achieved encapsulation of 25 ± 8 μg_GLU_/mg_LIP_ (*X*_c_ = 1.00). In contrast to the encapsulation of carboxyfluorescein, extrusion was no longer an effective approach. The film method followed by extrusion yielded low encapsulation of glucose (S/L ratio = 14 ± 4 μg_GLU_/mg_LIP_, *X*_c_ = 0.25) and even its further combination with freezing cycles did not result in a statistically significant improvement (S/L ratio = 17 ± 4 μg_GLU_/mg_LIP_, *X*_c_ = 0.30, *p* > 0.3).

These results indicate that the film hydration method with extrusion can be an effective approach for encapsulating compounds into liposomes. However, its efficiency significantly decreased with decreasing permeability of the substance. On the other hand, sonication did not suffer as much from the decreasing permeability of the substances and even showed increasing extent of encapsulation with increasing hydrophilicity of the encapsulated solute. The freeze–thawing method was the least effective among the tested methods. Its effectiveness also decreased with the decreasing permeability, and the only advantage was its ability to enhance the encapsulation of already loaded liposomes.

### 3.4 Discussion of Liposome Forming Mechanisms

A fundamental difference between the film hydration and heating method is based on the mechanism by which the primary assembly of the liposomes occurs. The film hydration method relies on solution penetration into vesicles budding from the lipid film in which preassembled lipid layers already exist from the solvent evaporation step. This membrane is highly curved and not in the thermodynamically final state; thus, it is more permeable for both water and solutes than the membrane in the already formed liposomes.^28,41^ The effectiveness of encapsulation of poorly permeable solutes can, therefore, be limited by membrane rejection (Figure 5A). In contrast, liposomes formed by the heating method are assumed to assemble around a pocket of the aqueous solution from highly disordered lipid fragments, theoretically preserving the original solute concentration in that region of the aqueous phase (Figure 5B). They tend to self-assemble into vesicles, where their size is determined by the balance between minimizing the surface area and the bending energy of the membrane, as discussed elsewhere.^42^ For the primary liposomes already formed, three mechanisms of improving the size and encapsulation efficiency of the already prepared liposomes were studied in this manuscript: extrusion, sonication, and freeze–thawing.

**Figure 5:**
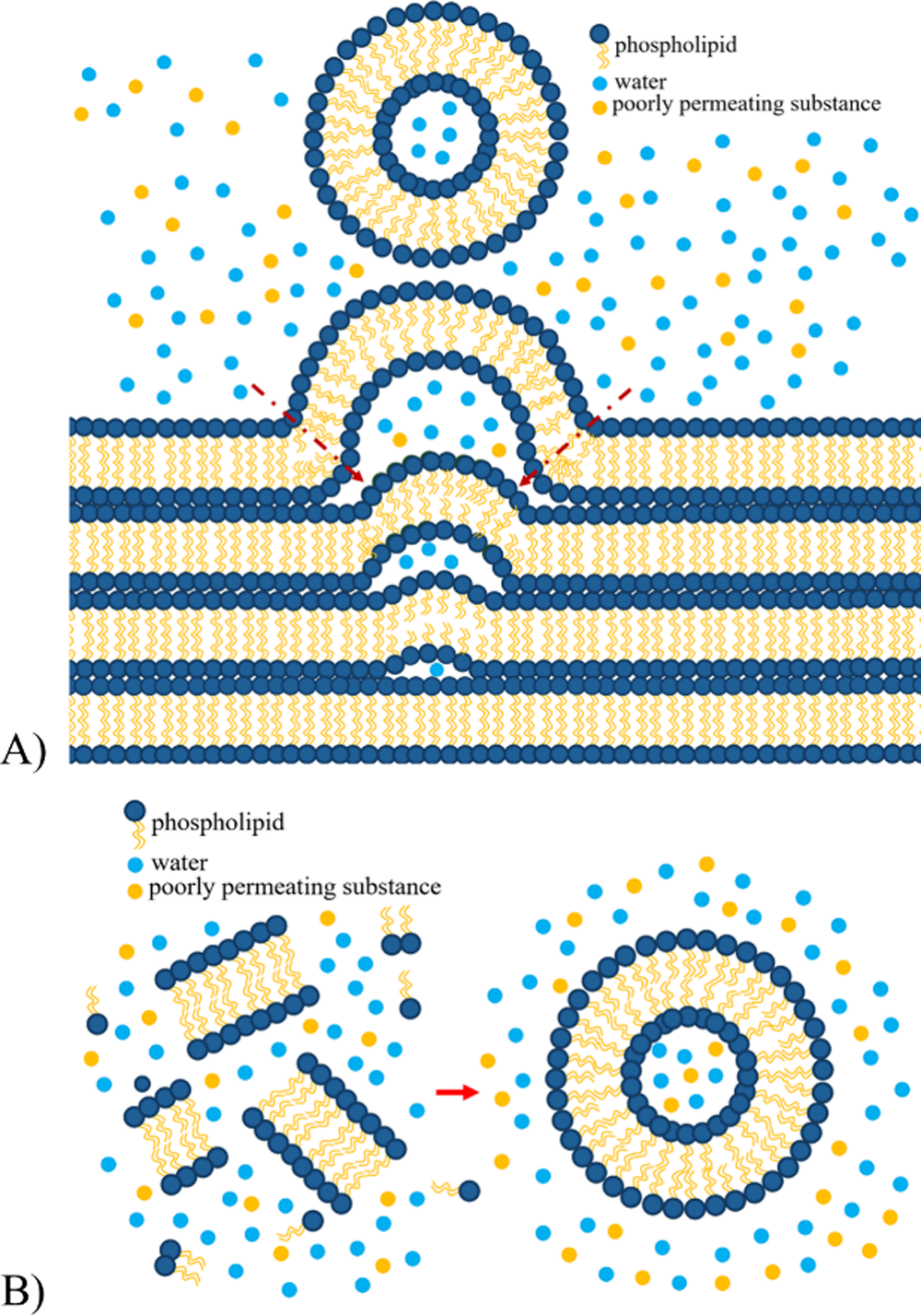
Schematic illustration of presumed liposome formation mechanisms by the lipid film hydration method (A) and by the heating method (B). In the film hydration method, poorly permeable solutes can be rejected by the budding lipid bilayer, while in the heating method, the liposome can hypothetically assemble around the solute.

When liposomes are prepared by extrusion, preformed multilamellar vesicles are forced through a polycarbonate membrane. This process, shown schematically in Figure 6, induces the transformation of larger vesicles into smaller ones, producing more uniform liposomes with sizes that correspond to the pore diameter of the extrusion membrane.^43^ As liposomes are pushed through the extrusion membrane, the lipid bilayer is repeatedly stretched, highly curved, and structurally disturbed, which can lead to increased solute permeation into the liposome core. However, as the bilayer stretching occurs mainly within the confined pore space of the extrusion membrane, we hypothesize that the local supply of the solute may be limited. While this limited local supply offers a plausible explanation for the observed lower encapsulation, verifying this mechanism would require further investigation.

**Figure 6:**
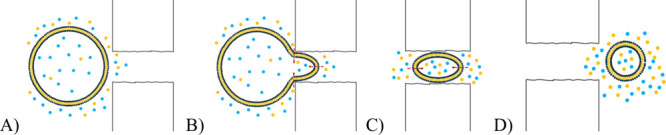
Schematic illustration of the assumed mechanism of liposome size adjustment by extrusion through a polycarbonate membrane. (A, B) Large parent vesicle entering a pore throat; (C) the vesicle is forced through the membrane pores, undergoing deformation, stretching, and rupture of the bilayer; (D) reassembly of daughter liposomes at the pore exit.

In the case of sonication, ultrasonic energy generates cavitation bubbles, which collapse violently and produce localized shear forces and pressure singularities. These forces can cause stretching, bulging, and fragmentation of the lipid bilayers of previously formed “raw” multilamellar vesicles, and the resulting fragments spontaneously reform into small unilamellar vesicles.^44^ This proposed mechanism is shown schematically in Figure 7. During this process, the solute can permeate from the bulk into the liposome core due to a temporary loss of lipid bilayer integrity. Thus, the solute can diffuse into the newly formed liposomes without a permeation barrier.

**Figure 7:**
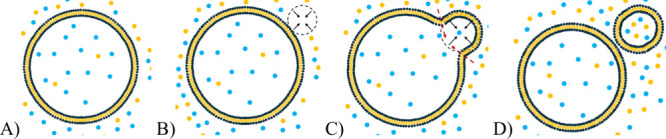
Schematic illustration of the assumed mechanism of the formation of small liposomes by sonication. (A) Large parent vesicle; (B) generation of a cavitation bubble in the vicinity of the vesicle due to ultrasound energy; (C) collapse of the cavitation bubble leading to bulging and fragmentation of the lipid bilayer; (D) reassembly of lipid bilayer fragments into a small daughter vesicle.

For the freezing–thawing, shown schematically in Figure 8, the mechanism of solute permeation enhancement has been reported in the literature.^45,46^ The freezing is assumed to form defects in the liposome membrane, and during thawing, the solute can diffuse through the temporary openings into the liposome core before the integrity of the lipid bilayer is restored at elevated temperatures.

**Figure 8:**
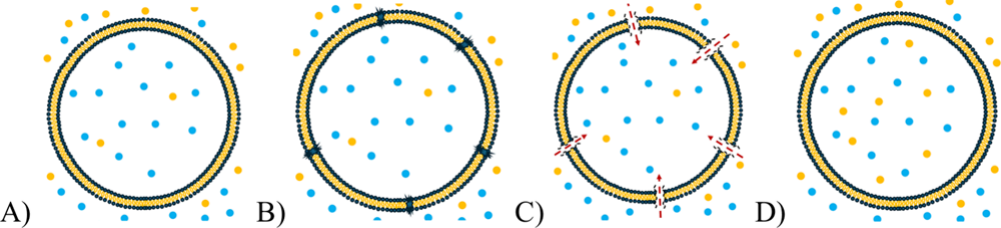
Schematic illustration of the assumed mechanism of solute encapsulation into liposomes during the freeze–thawing process. (A) Initial large vesicle; (B) formation of ice crystals disrupting the phospholipid bilayer during the freezing phase; (C) formation of temporary pores in the membrane during the thawing phase; (D) reassembly of the phospholipid bilayer.

### 3.5 Effect of Solute Concentration

If liposome loading occurs by the assembly of the lipid bilayer around a pocket of water containing the solute (as in the sonication process discussed above and shown in Figure 7), one might expect the encapsulated quantity to be directly proportional to the bulk solute concentration. On the other hand, if liposome loading were a permeation-driven process (as in the membrane extrusion process, shown in Figure 6), then the encapsulated quantity should also increase with the concentration driving force, but the intraliposomal concentration would be expected to be lower than that of the loading solution. To test these scenarios and to determine whether the encapsulated quantity is proportional to the solute concentration in the loading solution, experiments with a concentration series of 7.5, 25, 50, and 100 mg/mL were performed. These experiments were carried out with glucose thanks to its high solubility in water (for carboxyfluorescein, the previously used concentration of 7.5 mg/mL is already close to its maximum solubility limit).

The experimental data shown in Figure 9 reveal that increasing the solute concentration in the loading solution in a series from 7.5 to 25, 50, and 100 mg/mL indeed resulted in an increase in the encapsulated quantity for all three methods. However, regardless of the chosen liposome formulation process route, the increase of the S/L ratio with solute concentration was subproportional (regression analysis of the data provides a power-law dependence with exponents in the range of 0.6–0.7 with a high degree of accuracy, *R* ^2^ > 0.99, cf. Supporting Information). For comparison, Figure 9 also shows the theoretical encapsulated quantity that should be achieved if the intraliposomal concentration were equal to that of the loading solution. The liposomes in this theoretical calculation were assumed to be unilamellar, with an area per lipid of 65.7 Å^2^ and uniform diameter of either 50, 100, or 200 nm.

**Figure 9:**
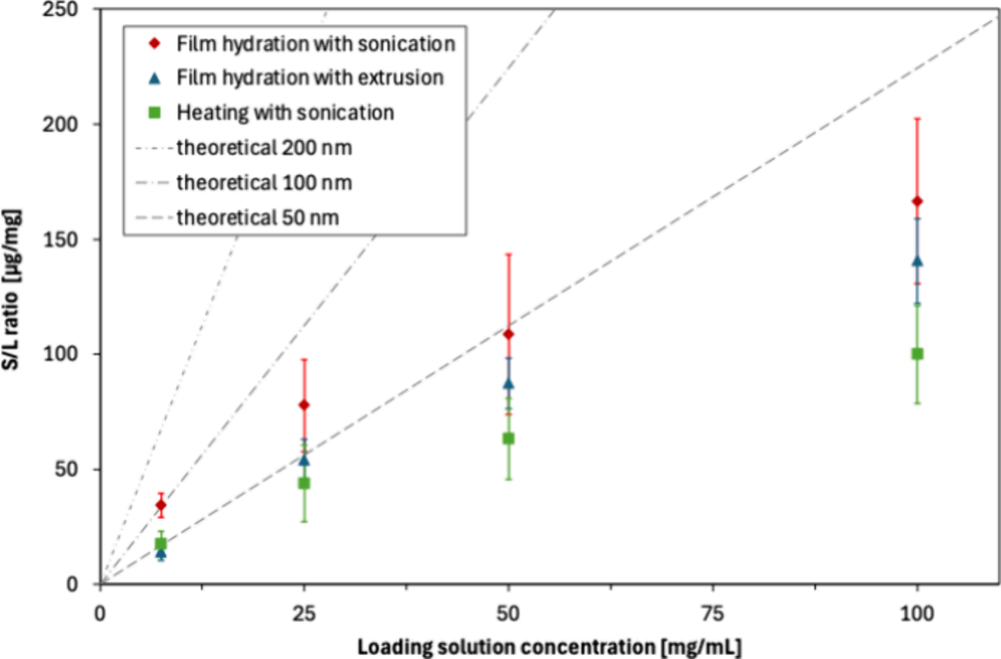
Effect of the process route and glucose concentration in the loading solution on the encapsulated quantity, expressed as the solute/lipid (S/L) ratio in the final liposomes (detailed experimental conditions are listed in Table 2). The data points represent mean values ± standard deviation (*N* = 3). The dashed lines represent theoretical S/L values that would be achieved in unilamellar liposomes of a given size if the intraliposomal glucose concentration were equal to that of the loading solution.

Let us recall from Figure 2 that liposomes processed by sonication had a volume-mean particle size around 60 nm, while those processed by extrusion had a volume-mean particle size around 170 nm. Thus, although liposomes formulated by the hydration–extrusion process route achieved an S/L ratio only approximately 20% lower than those formulated by the hydration-sonication process route, they lag significantly below their theoretical potential. On the other hand, the film hydration process followed by sonication appears to be rather efficient, providing solute loading at or only slightly below the theoretical limit. Liposomes formulated by the heating–sonication process route lag further behind, although this may be partially explained by the loss of approximately 30% of lipids as reported earlier. Both extrusion and sonication are assumed to result in temporary pore formation of the lipid bilayer, which should enable solute diffusion into the liposomal core even if the solute was initially rejected during the primary liposome formation step (cf. Figure 5). According to the “bilayer fragment” model,^47^ sonication disrupts lipidic structures through high-energy implosions, fragmenting the lipid bilayers into transient structures that immediately reassemble into SUVs. During membrane extrusion, the lipid bilayers are stretched rather than fragmented. In principle, both mechanisms of lipid bilayer disruption should provide an opportunity for solute diffusion from the bulk into the liposomal core to take place.

However, our experimental evidence suggests that liposome loading by solute diffusion occurred to a sufficient extent only during the sonication process and not during extrusion. This could be explained either by the duration of the bilayer disruption or by steric factors. Sonication results in cavitation events that can locally disrupt the liposomal bilayer. An imploding bubble can intensify local mass transfer, facilitation of the equilibration of intra- and extra-liposomal solute concentrations. In contrast, the stretching of the lipidic bilayer that occurs during the membrane extrusion process takes place inside the constrictions of a porous membrane, where the local supply of the solute might be limited for steric reasons. Consequently, the intraliposomal concentration achieved by this process lags behind that of the bulk solution. It can be concluded that poorly permeable solutes such as glucose can be loaded into liposomes quite successfully by using a sonication process, and the encapsulated quantity can be improved by increasing the solute concentration in the loading solution.

### 3.6 Study Limitations and Practical Implications

To ensure a direct comparison of factors driving vesicle assembly, the experimental design was limited to a single specific lipid composition (DPPC:DPPG:cholesterol at a 75:10:15 molar ratio) and two model solutes (5(6)-carboxyfluorescein and D-(+)-glucose). This standardization does not consider, for example, the influence of surface charges or lipid phase transition temperatures. Despite these limitations, the findings offer valuable guidelines for the process selection. First, efficient liposome postprocessing, specifically size reduction and lamellarity control, strictly requires operating temperatures above the phase transition temperature of the lipid mixture. Second, the choice of the preparation method dictates the final physicochemical properties. While sonication-based processes proved most effective for maximizing the loading of hydrophilic cargo, they resulted in broader size distributions. In contrast, extrusion provided superior control over polydispersity, ensuring a more uniform vesicle population.

Finally, this study provides a standardized data set essential for future rational process design. While currently limited to two model solutes, the expansion of this data set with additional molecules could facilitate the development of predictive algorithms. Specifically, such data could support the construction of decision trees capable of recommending the optimal preparation route and the final encapsulation solely on the basis of the input properties of the target API.

## 4 Conclusions

The present work investigated how the choice of the formulation process route affects the quality and encapsulation efficiency of liposomes loaded with a poorly permeable solute. Using two model substances, 5(6)-carboxyfluorescein and D-(+)-glucose, we compared five different process routes head-to-head, keeping all other parameters as consistent as possible. The film hydration process route with extrusion, which produced liposomes with well-controlled size and low polydispersity, was the most effective for encapsulating CF. However, the efficiency of this process route decreased for less permeable glucose, presumably due to solute rejection by the budding lipid bilayer during film hydration. The freeze–thaw process route was found to be the least effective approach overall. In contrast, sonication-based approaches were not so much affected by the decrease in permeability, presumably because solute entry into the liposome occurs due to a temporary loss of bilayer integrity during cavitation events. The film hydration process route with sonication even resulted in a higher encapsulation for glucose than for carboxyfluorescein. The heating method resulted in a heterogeneous population of liposomes and a considerable fraction of unused phospholipids. However, its scalability is a distinct advantage and the fraction of unused lipids could probably be reduced by further process optimization. Crucially, the present study has found that for the sonication-based process routes, solute encapsulation can be increased by increasing its concentration in the loading solution. Together, these findings can serve as a tool for process route selection for liposome-based formulations of poorly permeable actives.

## Supplementary Material



## Data Availability

Research data for this article are available on Zenodo at DOI: 10.5281/zenodo.17921139.

## Notes

Stability of liposomes in time, transmission electron microscopy (TEM) images of selected liposome samples along with quantitative evaluation of particle size from TEM, calibration curves for the UV–vis (glucose) and fluorescence (carboxyfluorescein) spectroscopic determination of solute concentrations, a table with the numerical data supporting Figure 9, and finally a chart showing a correlation between the solute/lipid ratio and the glucose concentration in the loading solution for three liposome loading methods (PDF)
