# Supplementary material for: Direct Experimental Comparison of Liposome Formulation Processes for Poorly Permeable Solutes: Effect of Driving Force and Bilayer Disruption Mechanism
Source: ACS Omega. 2026 Apr 16;11(17):25046–56. doi: 10.1021/acsomega.5c09893 (PMC13150605; doi:10.1021/acsomega.5c09893)
Supplement: Supplementary file 1 [file ao5c09893_si_001.pdf]

Supplementary Information

Direct experimental comparison of liposome formulation processes for poorly permeable solutes: Effect of driving force and bilayer disruption mechanism

Martin Roudný[1], Martin Balouch[1,2], Jaroslav Hanuš[1], František Štěpánek[1,*]

[1]Department of Chemical Engineering, University of Chemistry and Technology Prague, Technická 5, Prague 6 166 28, Czech Republic
[2]Zentiva, k.s., U Kabelovny 130, Prague 10 102 37, Czech Republic
[*]Corresponding author. E-mail: Frantisek.Stepanek@vscht.cz

Table S1: Numerical values for Figure 4 from the main manuscript on the stability of liposomes over 7 days of storage at 4 °C, prepared by different methods as indicated. Data represent the volume-mean particle size and the polydispersity index (PDI).

**Formulation process route**	**Day 0**	**Day 1**	**Day 4**	**Day 7**
Heating with sonication	74 nm (0.27)	81 nm (0.25)	68 nm (0.23)	72 nm (0.22)
Film method with extrusion	171 nm (0.07)	168 nm (0.08)	170 nm (0.08)	167 nm (0.07)
Film method with sonication	77 nm (0.33)	98 nm (0.35)	129 nm (0.44)	111 nm (0.27)

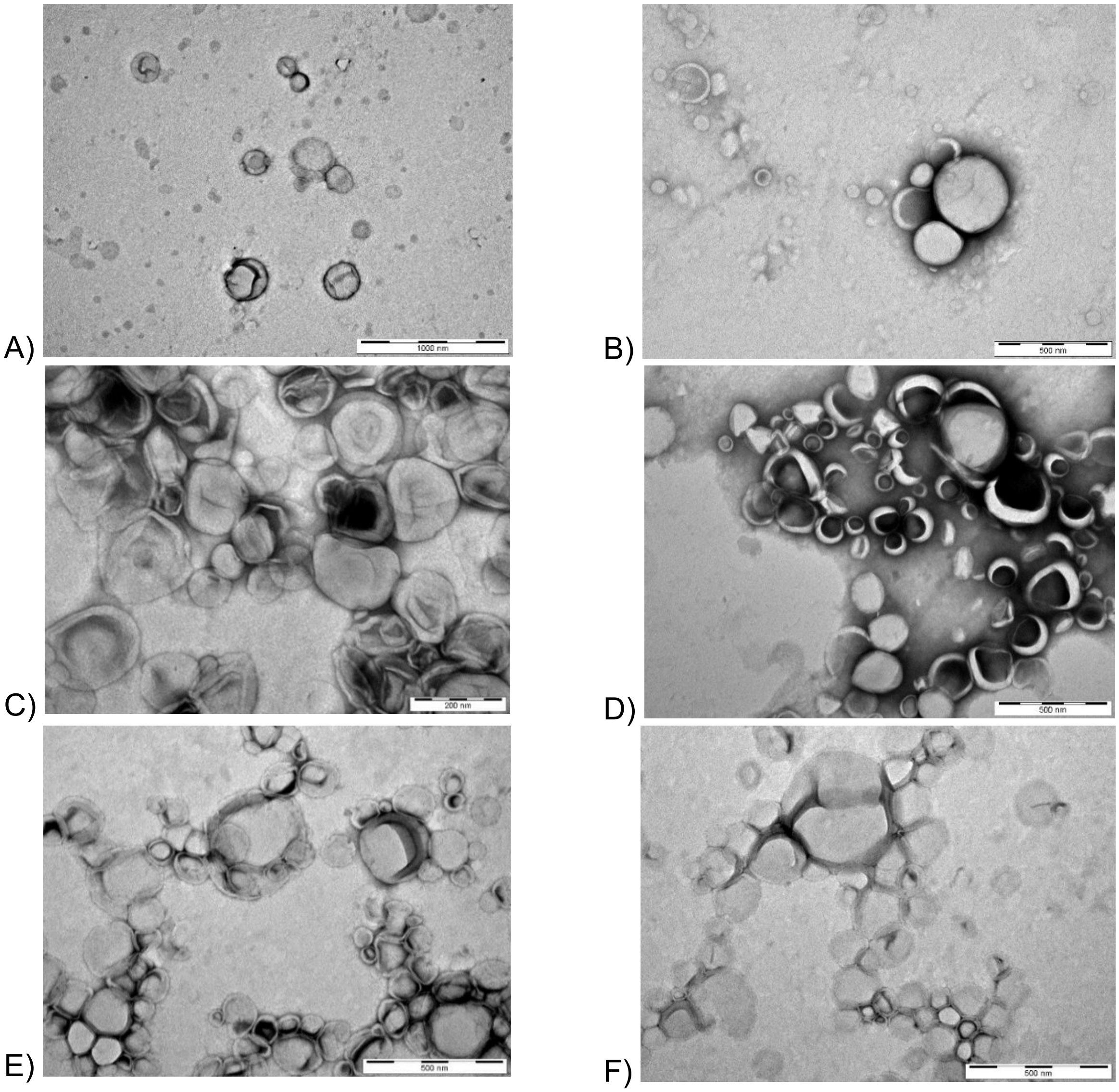

Figure S1: Additional representative transmission electron microscopy images of liposomes prepared by different process routes. (A, B) Liposomes prepared by the heating method followed by sonication. (C, D) Liposomes prepared by the film hydration method followed by extrusion. (E, F) Liposomes prepared by the film hydration method followed by sonication. Scale bars represent 200 nm (C), 500 nm (B,D,E, and F), 1000 nm (A).

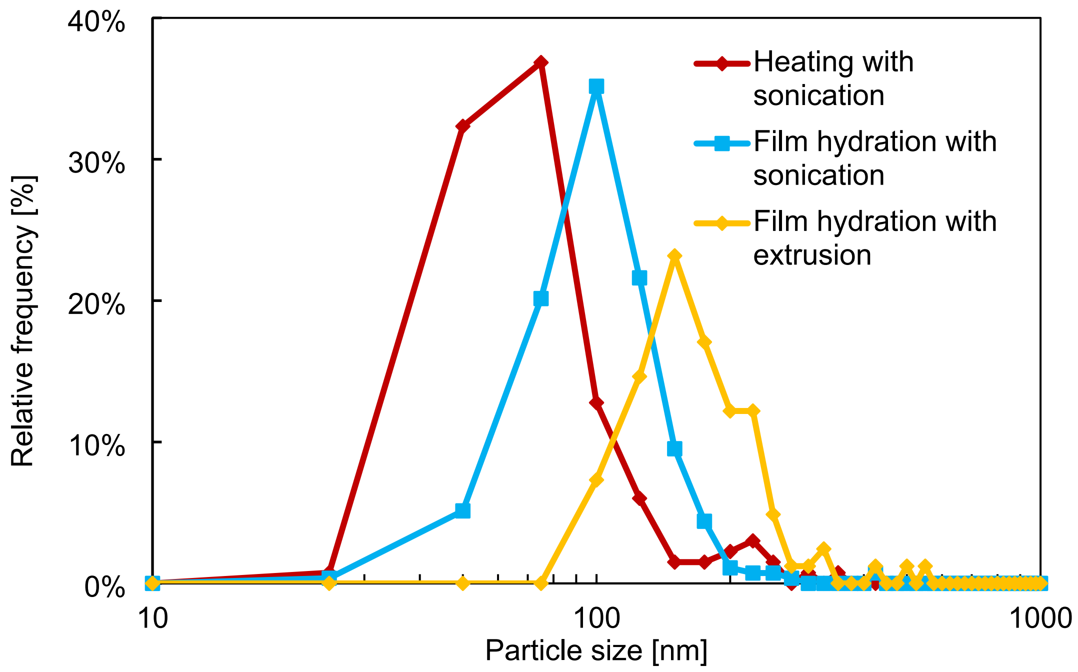

Figure S2. Quantitative particle size distribution derived from the analysis of TEM figures (corresponding to images in Figure 3 of the main text and Figure S1 from the Supplementary Information). Data were obtained by measuring the diameter of individual vesicles (n > 80) using ImageJ software.

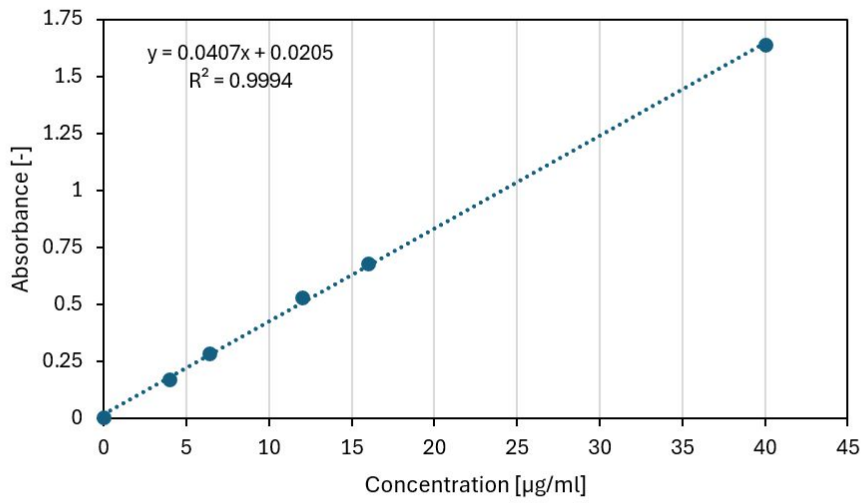

Figure S3. Calibration curve for D-(+)-glucose, determined by UV-VIS spectrophotometry at λ = 540 nm

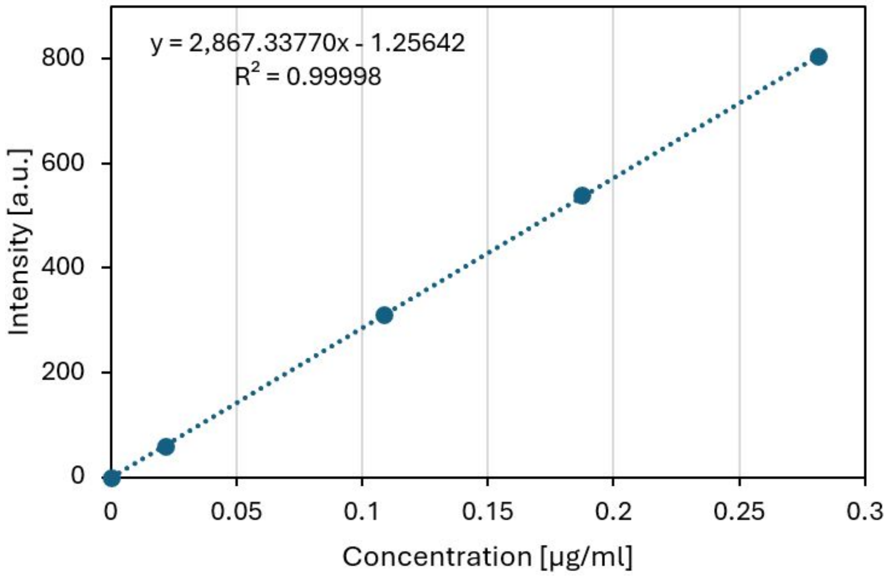

Figure S4. Calibration curve for 5(6)-carboxyfluorescein determined by fluorescence spectroscopy (λ_{ex} = 492 nm, λ_{em} = 517 nm) in PBS (pH 7.4)

Table S2. Numerical values of glucose encapsulation, presented in Fig. 9 of the main manuscript.

**Process route**	**Encapuslation for different loading concentration [$\mu g_{API}/mg_{LIP}$]**			
	**7.5 mg/ml**	**25 mg/ml**	**50 mg/ml**	**100 mg/ml**
Heating with sonication	18 ± 5	44 ± 17	63 ± 18	100 ± 21
Film hydration with extrusion	14 ± 4	54 ± 9	87 ± 11	141 ± 18
Film hydration with sonication	34 ± 5	78 ± 20	109 ± 35	166 ± 36

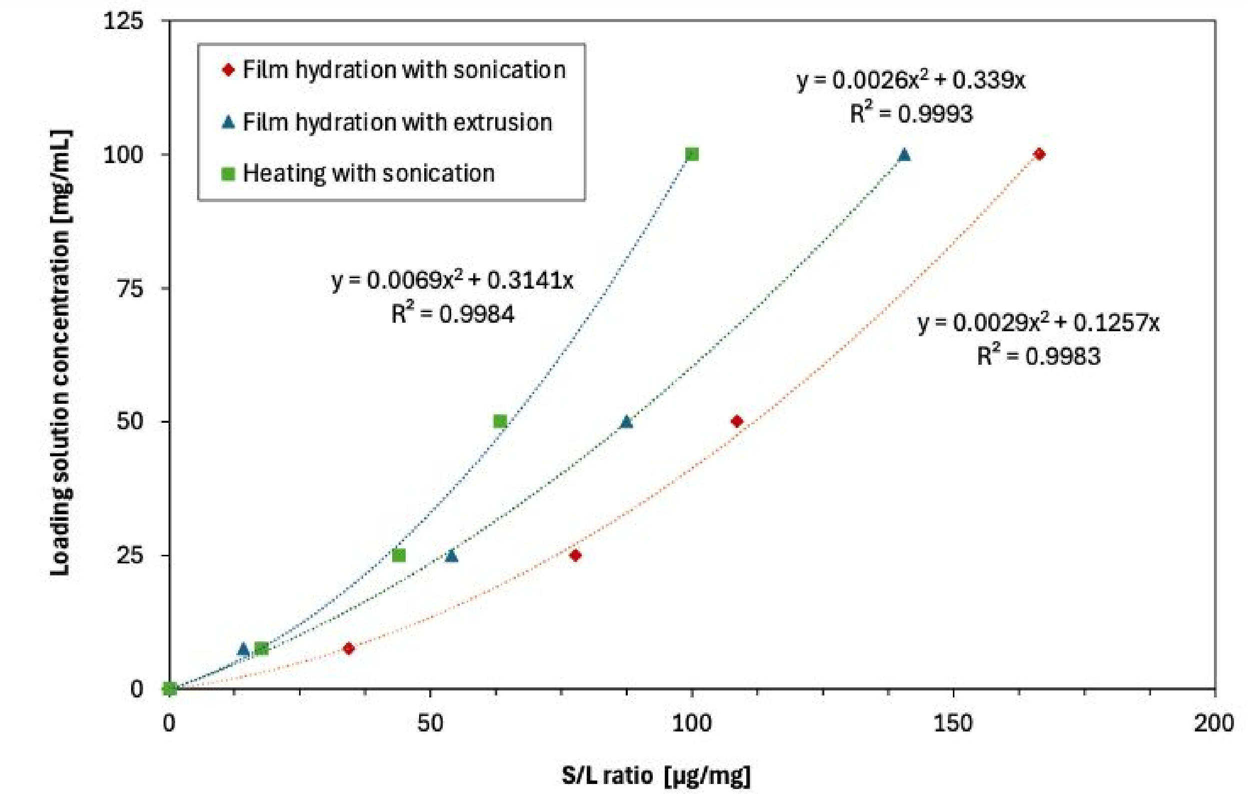

Figure S5. Regression analysis of glucose encapsulation data shown in Fig. 9 if the main manuscript.
